# *tert*-Butyl *N*-[(11-*exo*-benzyl­oxy­carbonyl-8-oxopenta­cyclo­[5.4.0.0^2,6^.0^3,10^.0^5,9^]undecane-11-*endo*-yloxy)carbon­yl­methyl]carbamate

**DOI:** 10.1107/S1600536810036627

**Published:** 2010-09-25

**Authors:** Rajshekhar Karpoormath, Thavendran Govender, Patrick Govender, Hendrik G. Kruger, Glenn E. M. Maguire

**Affiliations:** a School of Chemistry, University of KwaZulu–Natal, Durban 4000, South Africa; b School of Pharmacy and Pharmacology, University of KwaZulu–Natal, Durban, South Africa; c Department of Biochemistry, University of KwaZulu–Natal, Durban 4000, South Africa

## Abstract

The structure of the title compound, C_26_H_29_NO_7_, at 173 K has an inter­molecular N—H⋯O hydrogen bond. This is one of the few examples where a mono-ketone penta­cyclo­undecane (PCU) mol­ecule exibits hydrogen bonding in the solid state. The dihedral angles of the amide and ester groups are normal and unaffected by the cage structure. A longer than normal C—C bond [1.571 (4) Å] was found within the cage structure.

## Related literature

For examples of cage structures with C—C bonds lengths that differ from normal, see: Marchand (1989 ▶); Kruger *et al.* (2006 ▶). For examples of crystal structures of mono-ketone PCU mol­ecules bearing heteroatoms, see: Watson *et al.* (2000 ▶); Flippen-Anderson *et al.* (1991 ▶); Liu *et al.* (2001 ▶). For the synthesis of the precursors, see: Martins *et al.* (1993 ▶).
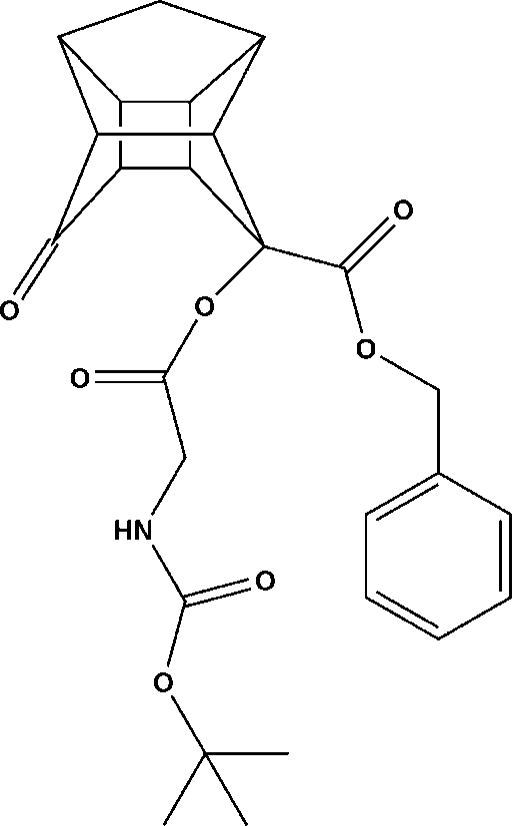


## Experimental

### 

#### Crystal data

C_26_H_29_NO_7_
*M* *_r_* = 467.50
Monoclinic, 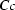

*a* = 9.7260 (2) Å
*b* = 27.5398 (7) Å
*c* = 9.3500 (2) Å
β = 107.679 (1)°
*V* = 2386.14 (9) Å^3^
*Z* = 4
Cu *K*α radiation
μ = 0.78 mm^−1^
*T* = 173 K
0.24 × 0.22 × 0.19 mm

#### Data collection

Bruker Kappa DUO APEXII diffractometer
Absorption correction: multi-scan (*SADABS*; Bruker, 2006 ▶) *T*_min_ = 0.694, *T*_max_ = 0.753
8919 measured reflections
3948 independent reflections
3802 reflections with *I* > 2σ(*I*)
*R*_int_ = 0.024

#### Refinement

*R*[*F* ^2^ > 2σ(*F* ^2^)] = 0.038
*wR*(*F* ^2^) = 0.100
*S* = 1.07
3948 reflections
311 parameters
3 restraints
H atoms treated by a mixture of independent and constrained refinement
Δρ_max_ = 0.21 e Å^−3^
Δρ_min_ = −0.18 e Å^−3^
Absolute structure: Flack (1983 ▶), 1760 Friedel pairs
Flack parameter: 0.00 (18)

### 

Data collection: *APEX2* (Bruker, 2006 ▶); cell refinement: *SAINT* (Bruker, 2006 ▶); data reduction: *SAINT*; program(s) used to solve structure: *SHELXS97* (Sheldrick, 2008 ▶); program(s) used to refine structure: *SHELXL97* (Sheldrick, 2008 ▶); molecular graphics: *X-SEED* (Barbour, 2001 ▶); software used to prepare material for publication: *SHELXL97*.

## Supplementary Material

Crystal structure: contains datablocks I, global. DOI: 10.1107/S1600536810036627/om2360sup1.cif

Structure factors: contains datablocks I. DOI: 10.1107/S1600536810036627/om2360Isup2.hkl

Additional supplementary materials:  crystallographic information; 3D view; checkCIF report

## Figures and Tables

**Table 1:** Hydrogen-bond geometry (Å, °)

*D*—H⋯*A*	*D*—H	H⋯*A*	*D*⋯*A*	*D*—H⋯*A*
N1—H1*N*⋯O1^i^	0.96 (1)	1.99 (2)	2.882 (2)	154 (2)

Symmetry code: (i) 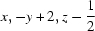.

**Fig. 1.**
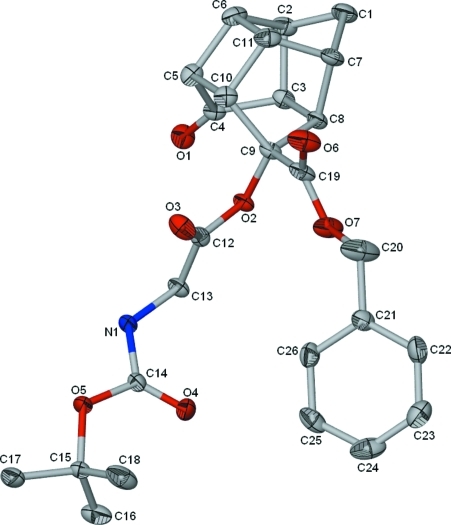
Molecular structure of the title compound showing the numbering scheme. All non-hydrogen atoms are shown as ellipsoids with probability level of 30%.

**Fig. 2.**
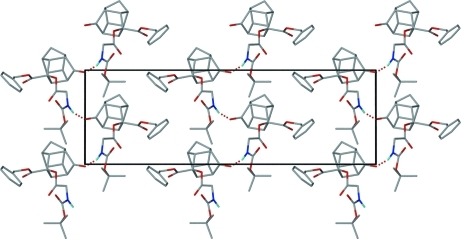
Partial projection viewed along [100]. All hydrogen atoms except the hydrogen H1N on N1 are omitted. The hydrogen bonds are shown as dotted lines.

## supplementary crystallographic information

### Comment

Pentacycloundecane (PCU) derivatives have been reported in a wide range of chemical fields (Marchand, 1989). We have reported the structures of a number of PCU derivatives including a mono-ketone ethylene acetal (Kruger *et al.*, 2006). Previous examples of this group of mono-ketone molecules have included crown ether (Watson *et al.*, 2000), nitro derivatives (Flippen-Anderson *et al.*, 1991) and benzylic amines (Liu *et al.*, 2001). The title compound (Fig. 1) is a rare example of a monoketone PCU with an intermolecular hydrogen bonding interaction and also features a PCU cage ester bond (Watson *et al.*, 2000). The intermolecular N1—H···O1 bond interaction (2.882 Å) holds the structure in interdigitated rows. The intermolecular distances between the ring centroids in the *a* axis direction of 5.641–7.710 Å, suggests that there is no π-stacking interaction between parallel molecules (Fig. 2).

### Experimental

5-Hydroxy-4-oxahexacyclo[5.4.0.0^2,6^.0^3,10^.0^5,9^]dodecane-3-carboxybenzylester (1.0 g, 3.2 mmol) (Martins *et al.*, 1993) was dissolved in dichloromethane (15 ml) and reacted, under stirring, with BOC-glycine (1.2 g, 6.4 mmol), diisopropylcarbodiimide (0.7 ml, 6.4 mmol), and DMAP (0.04 g, 0.32 mmol) overnight at ambient temperature. The resulting solution was washed with HCl (0.1 N) and water and then extracted with ethyl acetate. The organic layer was dried Na_2_SO_4_, filtered, and concentrated under vacuum. The product was isolated by silica flash column chromatography (15% ethyl acetate in hexane) to give the product, (0.6 g, 60% yield). Crystallization of the product was carried out by dissolving the product in 5 ml a solvent mixture of ethyl acetate and hexane (1:5) at 22 °C.

^1^H NMR (CDCl_3_, 400 MHz) δ p.p.m.: 1.42 (9.0H, s), 1.53 (1.0H, d, J=11.17 Hz), 1.72 (1.0H, s), 1.84 (1.0H, d, J=11.25 Hz), 2.49 (2.0H, m, J=2.21 Hz), 2.58 (1.0H, d, J=4.04 Hz), 2.63 (1.0H, d, J=3.72 Hz), 2.81 (1.0H, m, J=5.01 Hz), 2.99 (1.0H, q, J=3.19 Hz), 3.05 (1.0H, d, J=6.92 Hz), 3.59 (1.0H, t, J=7.06 Hz), 3.83 (2.0H, m, J=9.47 Hz), 4.85 (1.0H, s), 5.12 (2.0H, d, J=5.32 Hz), 7.28–7.34 (5.0H, m, J=4.99 Hz).

^13^C NMR (CDCl_3_, 100 MHz) δ p.p.m.: 28.32 (*s*), 36.25 (*s*), 38.15 (*s*), 40.54 (*s*), 41.50 (*s*), 41.93 (*s*), 42.35 (*s*), 43.15 (*s*), 43.92 (*s*), 50.17 (*s*), 54.33 (*s*), 67.42 (*s*), 79.87 (*s*), 84.66 (*s*), 128.43 (t, J=20.32 Hz), 135.10 (*s*), 155.41 (*s*), 169.43 (d, J=23.32 Hz), 214.23 (*s*)

IR (neat) V_max_ cm^-1^:3373.08, 2973.02, 1710.33, 1518.09, 1366.27, 1268.62, 1150.16, 1116.67, 1061.14, 743.63, 697.37.

HR ESI m/*z*: calcd for C_26_H_29_NO_7_ [*M*+H]^+^: 490.1826 found 490.1823.

### Refinement

All non-hydrogen atoms were refined anisotropically. All hydrogen atoms, except H1N on N1, were positioned geometrically with C—H = 0.95 - 1.00 Å and refined as riding on their parent atoms with *U*_iso_ (H) = 1.2 - 1.5 *U*_eq_ (C). The hydrogen atom H1N was located in a difference electron density maps and refined with simple bond length constraints.

### Crystal data

**Table:**

C_26_H_29_NO_7_	*F*(000) = 992
*M**_r_* = 467.50	*D*_x_ = 1.301 Mg m^−^^3^
Monoclinic, *C**c*	Cu *K*α radiation, λ = 1.54178 Å
*a* = 9.7260 (2) Å	Cell parameters from 8919 reflections
*b* = 27.5398 (7) Å	θ = 5.9–68.1°
*c* = 9.3500 (2) Å	µ = 0.78 mm^−^^1^
β = 107.679 (1)°	*T* = 173 K
*V* = 2386.14 (9) Å^3^	Block, colourless
*Z* = 4	0.24 × 0.22 × 0.19 mm

### Data collection

**Table:**

Bruker Kappa DUO APEXII diffractometer	3948 independent reflections
Radiation source: fine-focus sealed tube	3802 reflections with *I* > 2σ(*I*)
graphite	*R*_int_ = 0.024
0.5° φ scans and ω scans	θ_max_ = 68.1°, θ_min_ = 5.9°
Absorption correction: multi-scan (*SADABS*; Bruker, 2006)	*h* = −11→11
*T*_min_ = 0.694, *T*_max_ = 0.753	*k* = −30→32
8919 measured reflections	*l* = −11→10

### Refinement

**Table:**

Refinement on *F*^2^	Secondary atom site location: difference Fourier map
Least-squares matrix: full	Hydrogen site location: inferred from neighbouring sites
*R*[*F*^2^ > 2σ(*F*^2^)] = 0.038	H atoms treated by a mixture of independent and constrained refinement
*wR*(*F*^2^) = 0.100	*w* = 1/[σ^2^(*F*_o_^2^) + (0.0509*P*)^2^ + 1.0452*P*] where *P* = (*F*_o_^2^ + 2*F*_c_^2^)/3
*S* = 1.07	(Δ/σ)_max_ = 0.001
3948 reflections	Δρ_max_ = 0.21 e Å^−^^3^
311 parameters	Δρ_min_ = −0.18 e Å^−^^3^
3 restraints	Absolute structure: Flack (1983), 1760 Friedel pairs
Primary atom site location: structure-invariant direct methods	Flack parameter: 0.00 (18)

### Special details

**Table:**

Experimental. Half sphere of data collected using *SAINT* strategy (Bruker, 2006). Crystal to detector distance = 50 mm; combination of φ and ω scans of 0.5°, 40 s per °, 2 iterations.
Geometry. All e.s.d.'s (except the e.s.d. in the dihedral angle between two l.s. planes) are estimated using the full covariance matrix. The cell e.s.d.'s are taken into account individually in the estimation of e.s.d.'s in distances, angles and torsion angles; correlations between e.s.d.'s in cell parameters are only used when they are defined by crystal symmetry. An approximate (isotropic) treatment of cell e.s.d.'s is used for estimating e.s.d.'s involving l.s. planes.
Refinement. Refinement of *F*^2^ against ALL reflections. The weighted *R*-factor *wR* and goodness of fit *S* are based on *F*^2^, conventional *R*-factors *R* are based on *F*, with *F* set to zero for negative *F*^2^. The threshold expression of *F*^2^ > σ(*F*^2^) is used only for calculating *R*-factors(gt) *etc*. and is not relevant to the choice of reflections for refinement. *R*-factors based on *F*^2^ are statistically about twice as large as those based on *F*, and *R*- factors based on ALL data will be even larger.

### Fractional atomic coordinates and isotropic or equivalent isotropic displacement parameters (Å^2^)

**Table:**

	*x*	*y*	*z*	*U*_iso_*/*U*_eq_	
O1	0.65693 (19)	1.00052 (6)	0.9771 (2)	0.0521 (4)	
O2	0.76432 (15)	0.90113 (5)	0.91549 (15)	0.0358 (3)	
O3	0.7061 (2)	0.87716 (7)	0.67501 (18)	0.0564 (5)	
O4	1.1178 (2)	0.90102 (8)	0.6733 (2)	0.0619 (5)	
O5	1.00520 (16)	0.93227 (6)	0.44177 (16)	0.0452 (4)	
O6	0.5895 (2)	0.78765 (6)	0.8670 (3)	0.0661 (5)	
O7	0.8234 (2)	0.80702 (6)	0.9488 (3)	0.0642 (5)	
N1	0.9078 (2)	0.94186 (8)	0.6227 (2)	0.0454 (4)	
H1N	0.836 (2)	0.9596 (8)	0.549 (2)	0.049 (7)*	
C1	0.4838 (3)	0.88257 (10)	1.2375 (3)	0.0584 (6)	
H1A	0.5564	0.8807	1.3379	0.070*	
H1B	0.3871	0.8739	1.2442	0.070*	
C2	0.4852 (3)	0.93103 (9)	1.1615 (3)	0.0504 (6)	
H2	0.4599	0.9597	1.2140	0.060*	
C3	0.6376 (3)	0.93282 (8)	1.1385 (2)	0.0423 (5)	
H3	0.7140	0.9474	1.2248	0.051*	
C4	0.6031 (2)	0.96254 (8)	0.9971 (2)	0.0411 (5)	
C5	0.4713 (3)	0.93899 (8)	0.8917 (3)	0.0464 (5)	
H5	0.4163	0.9584	0.8021	0.056*	
C6	0.3875 (3)	0.92269 (9)	1.0015 (3)	0.0507 (6)	
H6	0.2846	0.9332	0.9777	0.061*	
C7	0.5258 (3)	0.85303 (8)	1.1207 (3)	0.0503 (6)	
H7	0.5335	0.8173	1.1397	0.060*	
C8	0.6645 (2)	0.87760 (8)	1.1087 (2)	0.0385 (4)	
H8	0.7545	0.8642	1.1809	0.046*	
C9	0.6564 (2)	0.86978 (7)	0.9442 (2)	0.0375 (4)	
C10	0.5005 (3)	0.88424 (8)	0.8639 (3)	0.0445 (5)	
H10	0.4605	0.8727	0.7579	0.053*	
C11	0.4164 (3)	0.86818 (9)	0.9720 (3)	0.0503 (6)	
H11	0.3305	0.8468	0.9305	0.060*	
C12	0.7770 (2)	0.90133 (7)	0.7760 (2)	0.0377 (4)	
C13	0.8940 (3)	0.93654 (8)	0.7718 (2)	0.0422 (5)	
H13A	0.8725	0.9686	0.8077	0.051*	
H13B	0.9870	0.9252	0.8409	0.051*	
C14	1.0199 (2)	0.92285 (8)	0.5870 (2)	0.0394 (5)	
C15	1.1196 (2)	0.91702 (8)	0.3776 (2)	0.0402 (5)	
C16	1.2606 (3)	0.94073 (13)	0.4606 (3)	0.0650 (8)	
H16A	1.2484	0.9761	0.4575	0.097*	
H16B	1.3340	0.9318	0.4131	0.097*	
H16C	1.2911	0.9297	0.5652	0.097*	
C17	1.0661 (3)	0.93591 (14)	0.2193 (3)	0.0681 (8)	
H17A	1.0606	0.9714	0.2210	0.102*	
H17B	0.9701	0.9225	0.1694	0.102*	
H17C	1.1328	0.9260	0.1644	0.102*	
C18	1.1285 (4)	0.86241 (11)	0.3777 (4)	0.0756 (9)	
H18A	1.0340	0.8489	0.3228	0.113*	
H18B	1.1574	0.8505	0.4814	0.113*	
H18C	1.2000	0.8523	0.3290	0.113*	
C19	0.6842 (3)	0.81669 (8)	0.9137 (3)	0.0483 (5)	
C20	0.8571 (4)	0.75648 (10)	0.9237 (6)	0.1008 (15)	
H20A	0.7839	0.7436	0.8334	0.121*	
H20B	0.8553	0.7364	1.0109	0.121*	
C21	1.0032 (3)	0.75425 (8)	0.9029 (3)	0.0489 (6)	
C22	1.0989 (4)	0.71945 (11)	0.9751 (3)	0.0657 (8)	
H22	1.0766	0.6993	1.0474	0.079*	
C23	1.2270 (3)	0.71335 (13)	0.9445 (4)	0.0726 (8)	
H23	1.2910	0.6882	0.9931	0.087*	
C24	1.2633 (3)	0.74254 (11)	0.8465 (5)	0.0752 (9)	
H24	1.3543	0.7389	0.8298	0.090*	
C25	1.1698 (4)	0.77737 (10)	0.7713 (3)	0.0625 (7)	
H25	1.1948	0.7976	0.7008	0.075*	
C26	1.0372 (3)	0.78318 (9)	0.7980 (3)	0.0537 (6)	
H26	0.9707	0.8069	0.7442	0.064*	

### Atomic displacement parameters (Å^2^)

**Table:**

	*U*^11^	*U*^22^	*U*^33^	*U*^12^	*U*^13^	*U*^23^
O1	0.0568 (10)	0.0371 (8)	0.0610 (10)	−0.0109 (7)	0.0157 (8)	−0.0025 (7)
O2	0.0419 (8)	0.0373 (7)	0.0348 (7)	−0.0062 (6)	0.0216 (6)	−0.0046 (6)
O3	0.0766 (12)	0.0561 (10)	0.0430 (9)	−0.0195 (9)	0.0279 (8)	−0.0168 (7)
O4	0.0599 (11)	0.0840 (13)	0.0476 (9)	0.0323 (9)	0.0251 (8)	0.0226 (9)
O5	0.0425 (8)	0.0613 (10)	0.0387 (8)	0.0141 (7)	0.0227 (7)	0.0115 (7)
O6	0.0722 (12)	0.0430 (10)	0.1014 (15)	−0.0175 (9)	0.0537 (12)	−0.0225 (9)
O7	0.0624 (11)	0.0325 (8)	0.1147 (15)	0.0020 (8)	0.0524 (11)	0.0000 (9)
N1	0.0460 (11)	0.0583 (11)	0.0396 (10)	0.0134 (9)	0.0244 (9)	0.0137 (8)
C1	0.0604 (16)	0.0588 (14)	0.0715 (16)	0.0052 (12)	0.0429 (14)	0.0033 (13)
C2	0.0528 (14)	0.0471 (13)	0.0614 (15)	−0.0017 (10)	0.0326 (12)	−0.0095 (11)
C3	0.0477 (12)	0.0437 (12)	0.0398 (11)	−0.0039 (9)	0.0200 (9)	−0.0083 (9)
C4	0.0410 (11)	0.0354 (11)	0.0498 (12)	−0.0035 (8)	0.0182 (10)	−0.0077 (9)
C5	0.0442 (12)	0.0386 (11)	0.0528 (13)	−0.0024 (9)	0.0095 (10)	−0.0039 (10)
C6	0.0374 (12)	0.0401 (12)	0.0777 (17)	0.0016 (9)	0.0219 (11)	−0.0018 (11)
C7	0.0553 (14)	0.0384 (11)	0.0744 (16)	0.0039 (10)	0.0455 (13)	0.0086 (10)
C8	0.0437 (11)	0.0375 (11)	0.0416 (11)	0.0001 (9)	0.0239 (9)	0.0002 (8)
C9	0.0419 (11)	0.0343 (10)	0.0436 (11)	−0.0072 (8)	0.0239 (9)	−0.0063 (8)
C10	0.0442 (12)	0.0392 (12)	0.0503 (12)	−0.0066 (9)	0.0148 (9)	−0.0098 (9)
C11	0.0432 (12)	0.0412 (12)	0.0724 (15)	−0.0093 (9)	0.0267 (11)	−0.0121 (11)
C12	0.0482 (12)	0.0341 (10)	0.0356 (10)	0.0030 (9)	0.0201 (9)	−0.0043 (8)
C13	0.0501 (12)	0.0475 (12)	0.0369 (11)	−0.0020 (9)	0.0248 (10)	−0.0016 (9)
C14	0.0435 (11)	0.0412 (11)	0.0381 (11)	0.0039 (9)	0.0193 (9)	0.0060 (9)
C15	0.0399 (11)	0.0477 (12)	0.0395 (11)	0.0057 (9)	0.0219 (9)	0.0026 (9)
C16	0.0528 (15)	0.090 (2)	0.0597 (16)	−0.0151 (14)	0.0291 (13)	−0.0203 (14)
C17	0.0548 (16)	0.113 (3)	0.0451 (13)	0.0158 (15)	0.0281 (12)	0.0128 (14)
C18	0.103 (2)	0.0515 (15)	0.101 (2)	0.0018 (15)	0.074 (2)	−0.0042 (15)
C19	0.0599 (14)	0.0373 (12)	0.0645 (14)	−0.0069 (10)	0.0441 (12)	−0.0068 (10)
C20	0.105 (3)	0.0329 (14)	0.207 (5)	0.0056 (14)	0.112 (3)	0.0042 (19)
C21	0.0586 (14)	0.0324 (10)	0.0659 (15)	0.0048 (10)	0.0341 (12)	0.0034 (10)
C22	0.090 (2)	0.0604 (16)	0.0514 (14)	0.0184 (15)	0.0279 (14)	0.0185 (12)
C23	0.0599 (17)	0.073 (2)	0.077 (2)	0.0147 (14)	0.0076 (15)	0.0090 (15)
C24	0.0459 (13)	0.0561 (17)	0.128 (3)	−0.0112 (13)	0.0331 (16)	−0.0265 (18)
C25	0.089 (2)	0.0453 (13)	0.0697 (17)	−0.0167 (13)	0.0478 (16)	−0.0030 (12)
C26	0.0608 (15)	0.0406 (12)	0.0575 (14)	0.0066 (10)	0.0144 (12)	0.0122 (10)

### Geometric parameters (Å, °)

**Table:**

O1—C4	1.210 (3)	C9—C10	1.527 (3)
O2—C12	1.347 (2)	C9—C19	1.529 (3)
O2—C9	1.445 (2)	C10—C11	1.546 (3)
O3—C12	1.189 (3)	C10—H10	1.0000
O4—C14	1.205 (3)	C11—H11	1.0000
O5—C14	1.347 (3)	C12—C13	1.505 (3)
O5—C15	1.476 (2)	C13—H13A	0.9900
O6—C19	1.198 (3)	C13—H13B	0.9900
O7—C19	1.320 (3)	C15—C16	1.504 (4)
O7—C20	1.465 (3)	C15—C17	1.505 (3)
N1—C14	1.340 (3)	C15—C18	1.506 (4)
N1—C13	1.448 (3)	C16—H16A	0.9800
N1—H1N	0.955 (10)	C16—H16B	0.9800
C1—C7	1.514 (4)	C16—H16C	0.9800
C1—C2	1.514 (4)	C17—H17A	0.9800
C1—H1A	0.9900	C17—H17B	0.9800
C1—H1B	0.9900	C17—H17C	0.9800
C2—C6	1.526 (4)	C18—H18A	0.9800
C2—C3	1.562 (3)	C18—H18B	0.9800
C2—H2	1.0000	C18—H18C	0.9800
C3—C4	1.504 (3)	C20—C21	1.494 (4)
C3—C8	1.582 (3)	C20—H20A	0.9900
C3—H3	1.0000	C20—H20B	0.9900
C4—C5	1.506 (3)	C21—C22	1.364 (4)
C5—C6	1.559 (4)	C21—C26	1.379 (3)
C5—C10	1.570 (3)	C22—C23	1.371 (5)
C5—H5	1.0000	C22—H22	0.9500
C6—C11	1.567 (3)	C23—C24	1.343 (5)
C6—H6	1.0000	C23—H23	0.9500
C7—C11	1.530 (4)	C24—C25	1.361 (5)
C7—C8	1.544 (3)	C24—H24	0.9500
C7—H7	1.0000	C25—C26	1.395 (4)
C8—C9	1.531 (3)	C25—H25	0.9500
C8—H8	1.0000	C26—H26	0.9500
			
C12—O2—C9	117.85 (16)	C7—C11—H11	117.4
C14—O5—C15	119.49 (17)	C10—C11—H11	117.4
C19—O7—C20	114.5 (2)	C6—C11—H11	117.4
C14—N1—C13	121.39 (19)	O3—C12—O2	124.4 (2)
C14—N1—H1N	119.6 (17)	O3—C12—C13	126.89 (19)
C13—N1—H1N	119.0 (17)	O2—C12—C13	108.69 (17)
C7—C1—C2	95.3 (2)	N1—C13—C12	112.50 (19)
C7—C1—H1A	112.7	N1—C13—H13A	109.1
C2—C1—H1A	112.7	C12—C13—H13A	109.1
C7—C1—H1B	112.7	N1—C13—H13B	109.1
C2—C1—H1B	112.7	C12—C13—H13B	109.1
H1A—C1—H1B	110.2	H13A—C13—H13B	107.8
C1—C2—C6	103.6 (2)	O4—C14—N1	124.4 (2)
C1—C2—C3	103.7 (2)	O4—C14—O5	126.3 (2)
C6—C2—C3	101.79 (18)	N1—C14—O5	109.31 (19)
C1—C2—H2	115.3	O5—C15—C16	110.40 (19)
C6—C2—H2	115.3	O5—C15—C17	102.88 (18)
C3—C2—H2	115.3	C16—C15—C17	110.2 (2)
C4—C3—C2	99.81 (19)	O5—C15—C18	109.52 (19)
C4—C3—C8	111.90 (17)	C16—C15—C18	113.0 (2)
C2—C3—C8	102.04 (17)	C17—C15—C18	110.4 (2)
C4—C3—H3	113.9	C15—C16—H16A	109.5
C2—C3—H3	113.9	C15—C16—H16B	109.5
C8—C3—H3	113.9	H16A—C16—H16B	109.5
O1—C4—C3	128.0 (2)	C15—C16—H16C	109.5
O1—C4—C5	126.4 (2)	H16A—C16—H16C	109.5
C3—C4—C5	105.04 (19)	H16B—C16—H16C	109.5
C4—C5—C6	101.77 (19)	C15—C17—H17A	109.5
C4—C5—C10	111.17 (19)	C15—C17—H17B	109.5
C6—C5—C10	89.40 (18)	H17A—C17—H17B	109.5
C4—C5—H5	116.8	C15—C17—H17C	109.5
C6—C5—H5	116.8	H17A—C17—H17C	109.5
C10—C5—H5	116.8	H17B—C17—H17C	109.5
C2—C6—C5	107.93 (19)	C15—C18—H18A	109.5
C2—C6—C11	103.0 (2)	C15—C18—H18B	109.5
C5—C6—C11	90.14 (18)	H18A—C18—H18B	109.5
C2—C6—H6	117.3	C15—C18—H18C	109.5
C5—C6—H6	117.3	H18A—C18—H18C	109.5
C11—C6—H6	117.3	H18B—C18—H18C	109.5
C1—C7—C11	104.1 (2)	O6—C19—O7	125.0 (2)
C1—C7—C8	104.5 (2)	O6—C19—C9	123.1 (2)
C11—C7—C8	101.31 (18)	O7—C19—C9	111.9 (2)
C1—C7—H7	115.1	O7—C20—C21	109.1 (2)
C11—C7—H7	115.1	O7—C20—H20A	109.9
C8—C7—H7	115.1	C21—C20—H20A	109.9
C9—C8—C7	103.44 (18)	O7—C20—H20B	109.9
C9—C8—C3	110.57 (17)	C21—C20—H20B	109.9
C7—C8—C3	102.31 (17)	H20A—C20—H20B	108.3
C9—C8—H8	113.2	C22—C21—C26	118.9 (3)
C7—C8—H8	113.2	C22—C21—C20	119.8 (3)
C3—C8—H8	113.2	C26—C21—C20	120.8 (3)
O2—C9—C10	114.94 (17)	C21—C22—C23	120.6 (3)
O2—C9—C19	110.94 (17)	C21—C22—H22	119.7
C10—C9—C19	111.30 (18)	C23—C22—H22	119.7
O2—C9—C8	106.48 (16)	C24—C23—C22	120.8 (3)
C10—C9—C8	101.39 (17)	C24—C23—H23	119.6
C19—C9—C8	111.31 (18)	C22—C23—H23	119.6
C9—C10—C11	104.35 (19)	C23—C24—C25	120.1 (3)
C9—C10—C5	111.82 (18)	C23—C24—H24	119.9
C11—C10—C5	90.48 (18)	C25—C24—H24	119.9
C9—C10—H10	115.7	C24—C25—C26	119.8 (3)
C11—C10—H10	115.7	C24—C25—H25	120.1
C5—C10—H10	115.7	C26—C25—H25	120.1
C7—C11—C10	108.19 (19)	C21—C26—C25	119.7 (2)
C7—C11—C6	102.3 (2)	C21—C26—H26	120.2
C10—C11—C6	89.98 (18)	C25—C26—H26	120.2
			
C7—C1—C2—C6	52.8 (2)	C4—C5—C10—C9	−2.9 (3)
C7—C1—C2—C3	−53.2 (2)	C6—C5—C10—C9	−105.3 (2)
C1—C2—C3—C4	149.0 (2)	C4—C5—C10—C11	102.8 (2)
C6—C2—C3—C4	41.6 (2)	C6—C5—C10—C11	0.38 (19)
C1—C2—C3—C8	33.9 (2)	C1—C7—C11—C10	127.0 (2)
C6—C2—C3—C8	−73.4 (2)	C8—C7—C11—C10	18.7 (2)
C2—C3—C4—O1	122.5 (3)	C1—C7—C11—C6	32.9 (2)
C8—C3—C4—O1	−130.2 (2)	C8—C7—C11—C6	−75.4 (2)
C2—C3—C4—C5	−49.2 (2)	C9—C10—C11—C7	9.3 (2)
C8—C3—C4—C5	58.2 (2)	C5—C10—C11—C7	−103.4 (2)
O1—C4—C5—C6	−136.1 (2)	C9—C10—C11—C6	112.32 (18)
C3—C4—C5—C6	35.7 (2)	C5—C10—C11—C6	−0.38 (18)
O1—C4—C5—C10	129.9 (3)	C2—C6—C11—C7	0.6 (2)
C3—C4—C5—C10	−58.2 (2)	C5—C6—C11—C7	109.02 (19)
C1—C2—C6—C5	−128.2 (2)	C2—C6—C11—C10	−108.1 (2)
C3—C2—C6—C5	−20.8 (2)	C5—C6—C11—C10	0.38 (19)
C1—C2—C6—C11	−33.8 (2)	C9—O2—C12—O3	−0.6 (3)
C3—C2—C6—C11	73.7 (2)	C9—O2—C12—C13	179.57 (17)
C4—C5—C6—C2	−8.2 (2)	C14—N1—C13—C12	−107.3 (3)
C10—C5—C6—C2	103.3 (2)	O3—C12—C13—N1	4.5 (3)
C4—C5—C6—C11	−111.89 (19)	O2—C12—C13—N1	−175.63 (18)
C10—C5—C6—C11	−0.38 (18)	C13—N1—C14—O4	−1.3 (4)
C2—C1—C7—C11	−52.6 (2)	C13—N1—C14—O5	178.9 (2)
C2—C1—C7—C8	53.3 (2)	C15—O5—C14—O4	−2.7 (4)
C1—C7—C8—C9	−148.1 (2)	C15—O5—C14—N1	176.98 (19)
C11—C7—C8—C9	−40.1 (2)	C14—O5—C15—C16	−59.9 (3)
C1—C7—C8—C3	−33.1 (2)	C14—O5—C15—C17	−177.5 (2)
C11—C7—C8—C3	74.8 (2)	C14—O5—C15—C18	65.1 (3)
C4—C3—C8—C9	3.2 (2)	C20—O7—C19—O6	1.1 (4)
C2—C3—C8—C9	109.08 (19)	C20—O7—C19—C9	179.3 (3)
C4—C3—C8—C7	−106.4 (2)	O2—C9—C19—O6	−147.2 (2)
C2—C3—C8—C7	−0.6 (2)	C10—C9—C19—O6	−17.9 (3)
C12—O2—C9—C10	−67.7 (2)	C8—C9—C19—O6	94.5 (3)
C12—O2—C9—C19	59.7 (2)	O2—C9—C19—O7	34.5 (3)
C12—O2—C9—C8	−179.06 (17)	C10—C9—C19—O7	163.9 (2)
C7—C8—C9—O2	167.11 (17)	C8—C9—C19—O7	−83.8 (2)
C3—C8—C9—O2	58.2 (2)	C19—O7—C20—C21	155.9 (3)
C7—C8—C9—C10	46.57 (19)	O7—C20—C21—C22	134.8 (3)
C3—C8—C9—C10	−62.3 (2)	O7—C20—C21—C26	−52.9 (5)
C7—C8—C9—C19	−71.9 (2)	C26—C21—C22—C23	0.2 (5)
C3—C8—C9—C19	179.23 (18)	C20—C21—C22—C23	172.6 (3)
O2—C9—C10—C11	−148.11 (17)	C21—C22—C23—C24	2.3 (5)
C19—C9—C10—C11	84.7 (2)	C22—C23—C24—C25	−2.9 (5)
C8—C9—C10—C11	−33.7 (2)	C23—C24—C25—C26	1.1 (5)
O2—C9—C10—C5	−51.7 (3)	C22—C21—C26—C25	−2.0 (4)
C19—C9—C10—C5	−178.88 (19)	C20—C21—C26—C25	−174.3 (3)
C8—C9—C10—C5	62.7 (2)	C24—C25—C26—C21	1.4 (4)

### Hydrogen-bond geometry (Å, °)

**Table:**

*D*—H···*A*	*D*—H	H···*A*	*D*···*A*	*D*—H···*A*
N1—H1N···O1^i^	0.96 (1)	1.99 (2)	2.882 (2)	154 (2)

Symmetry codes: (i) *x*, −*y*+2, *z*−1/2.
